# Editorial: Quantification of immunological memory

**DOI:** 10.3389/fimmu.2023.1219067

**Published:** 2023-06-05

**Authors:** Francesca Di Rosa, Filippo Castiglione, Uri Hershberg, Antonella Prisco

**Affiliations:** ^1^Institute of Molecular Biology and Pathology, Department of Biomedical Sciences, National Research Council (CNR), Rome, Italy; ^2^Biotech Research Center, Technology Innovation Institute, Masdar City, Abu Dhabi, United Arab Emirates; ^3^Department of Human Biology, Faculty of Sciences University of Haifa, Haifa, Israel; ^4^Institute of Genetics and Biophysics Adriano Buzzati-Traverso, Department of Biomedical Sciences, National Research Council (CNR), Naples, Italy

**Keywords:** recall memory, anamnestic response, memory persistence, B cell memory, T cell memory, modeling, vaccine

Quantification of immunological memory requires a diversity of approaches to accurately collect data on all the relevant cell types and molecules, identify the correlates of protection, and model the kinetics of the immune response. This collection of articles reports studies performed in humans, in mouse, and *in silico* models, that altogether emphasize the strength of integrating diverse methodologies for investigating the complexity of immunological memory.

## 1 Memory lymphocyte subtypes

Several contributors to this topic emphasized memory T and B cell heterogeneity. Humphries et al. offered a comprehensive overview of non-human primate and human studies on pulmonary-Resident Memory T (T_RM_) and B (B_RM_) lymphocytes in respiratory infections. These authors highlighted the need for improving vaccination strategies to induce T_RM_ and B_RM_, and methods to quantify these cells; they also discussed some promising novelties in the techniques to assess respiratory airways immunity. Yordanova et al. investigated memory T cell heterogeneity in recall responses to *H. polygyrus*, a strictly enteric nematode, in a mouse model. The peculiarity of this model is that memory T cells were found in *H. polygyrus*-cured mice in the peritoneal cavity and lungs, in addition to intestinal lamina propria and mesenteric lymph nodes (LNs). Distinct transcriptional profiles characterized T cells from each tissue. Upon *H. polygyrus* challenge, parasite-specific OX40^+^ Th2 cells expanded as early as day 3 in the peritoneum, coincident with the presence of abundant OX40L^+^ dendritic cells and eosinophils in this organ, while Th2 cells increased only at day 14 in mesenteric LNs.

The memory T cell compartment comprises both foreign antigen (Ag)-specific “authentic” memory and memory-phenotype (MP) cells, whose function is unclear. Kawabe et al. identified a set of markers distinguishing Ag-specific from MP cells in the mouse CD44hi CD62Llow memory CD4^+^ T cell compartment. Thus, the “authentic” memory cells, that in this study were elicited by infection with lymphocytic choriomeningitis virus (LCMV), were mostly CD127hi Sca1hi, while the MP cells were more heterogeneous in terms of CD127 and Sca1 expression. Notably, Bcl2 expression was significantly higher in CD127hi Sca1hi Ag-specific cells than in CD127hi Sca1hi MP cells. Further experiments demonstrated that CD127hi Sca1hi MP cells had a Th1-signature, consistent with a pro-inflammatory ‘innate-type’ function that can either protect at early times after encounter with a new Ag, before the development of Ag-specific immunity, or mediate immunopathology.

## 2 Ag receptor-repertoires and Ag-specificity of memory lymphocytes

Huisman et al. sequenced the TCR-repertoires of 190 purified memory CD8 T-cell populations from 29 healthy human donors, directed against 21 epitopes of Cytomegalovirus, Epstein-Barr virus, and Adenovirus, looking for so-called “public” TCR sequences shared among different individuals. The authors found that a large part of the sequenced TCRs contained Identical (I-PUB) and Highly Similar (HS-PUB) CDR3β, confirmed their results in an independent cohort, and proposed that the shared TCR sequences may be attractive candidates for innovative anti-viral therapies, e.g., based on TCR gene transfer. Although “public” TCRs may derive from a subset of memory T cells selected for their ability to control viral reactivation in chronic infections, they have been identified also in the TCR-repertoires of T cells specific for non-latent viruses. Ohm-Laursen et al. used Adaptive Immune Receptor Repertoire sequencing (AIRR-seq) to analyse the Ig repertoire from the respiratory tract of atopic asthma patients and controls and to investigate B cell clone dissemination in the airways. This study reported that atopic asthma patients had distinct Ig repertoires, and that B cell clones trafficked more prominently from the nasal to the bronchial mucosa, than between right and left bronchial trees. Furthermore, heavily mutated IgD-only B cells were found in the asthmatic bronchial mucosa, and their Ig sequences were consistent with the hypothesis of bacteria/superantigen-driven stimulation (e.g. in terms of IGHV-genes, CDR3 length, etc.). Neuman et al. presented IgTreeZ (Immunoglobulin Tree analyZer), a new tool to analyze Ig gene lineage trees and Ig repertoires, and showed that their method of lineage tree-based analysis was instrumental to account for mutations in the CDR3. For example, analysis of samples from COVID-19 patients by IgTreeZ demonstrated that extensive Ig somatic hypermutation (SHM) occurred between the second and fourth week after the onset of clinical symptoms, while affinity-maturated, structurally stable antibodies (Abs) appeared at about one month from clinical symptom onset.

The persistence of virus-specific Abs and their potential cross-reactions are topics of renewed interest in the context of COVID-19 pandemic. Tanunliong et al. performed a longitudinal study on the SARS-CoV-2 serostatus of long-term care residents living in a facility that experienced two COVID-19 outbreaks, one in April and the other in October 2020. The authors demonstrated persistence of Abs directed against the Spike Ag of SARS-CoV-2 over seven months, with a gradual waning of anti-Nucleocapsid Abs. They also found, among SARS-CoV-2 seropositive individuals, elevated Abs against human seasonal coronaviruses OC43 and HKU1, which may be attributed to a heterologous boosting effect by SARS-CoV-2 infection and/or cross-reaction.

## 3 Regulation of immunological memory

Immunological memory is generally associated with the concept of protective long-term immunity, e.g., post-vaccination, even though memory T cell responses may be insufficient or even detrimental in some diseases. Barnaba gave an extensive overview of the mechanisms supporting durable T cell memory and preventing excessive T cell activation and immunopathology, e.g., effector T cell exhaustion and suppression by regulatory T cells. The author critically discussed recent findings on immuno-regulatory cellular and molecular mechanisms, highlighting the remaining gaps, and proposing innovative strategies to improve T cell-mediated protection against infectious agents and cancer cells, and to inhibit the development and progression of autoimmune diseases. Using a network-based bioinformatics approach, Onisiforou and Spyrou described the effects of microbiota-host and microbiota-virus interactions on the regulation of immune response in neurodegenerative diseases. Their findings pointed out a marked impact of microbiota-mediated-immune effects on Multiple Sclerosis.

## 4 Vaccine-induced immunological memory

A better understanding of immunological memory can be highly beneficial to optimize vaccine formulations and vaccination protocols. Duhen et al. examined the extent to which OX40L:Ig, an OX40 agonist, enhanced T and B cell responses to either a protein and adjuvant-based or a self-amplifying mRNA-based vaccine against SARS-CoV-2 in mouse models. They tested OX40L:Ig according to different vaccination schemes (e.g., single or repeated OX40L:Ig injection, at the time of prime, boost, third booster, etc.) and found that it consistently increased both humoral and cellular responses. Natalini et al. investigated the impact of the time interval between vaccine doses on CD8 T cell immunity induced by prime with HIV-1 gag-encoding Chimpanzee adenovector and boost with HIV-1 gag-encoding Modified Vaccinia virus Ankara in a mouse model. A delayed boost (i.e., at day 100 post-prime) was more effective than an early one (i.e., at day 30 post-prime), as evaluated by multi-lymphoid organ assessment of gag-specific CD8 T cell frequency, cytotoxicity, and IFN-γ production. Cell number estimation showed that boost at day 100 yielded a ~3-fold higher number of gag-specific CD8 T cells in the sum of spleen and bone marrow (BM) than boost at day 30, and a ~15-fold higher number than prime only. The authors described a splenic memory CD8 T cell molecular signature associated with enhanced response to delayed boost that trended toward a central memory phenotype, and was characterized by shut off of several proliferative genes, and up-regulation of stem cell genes previously implicated in setting the equilibrium between quiescence and proliferation. Interestingly, gag-specific CD8 T cell frequency selectively diminished in the blood at day 100 post-prime, but not in the spleen, LNs, and BM, coincidently with the improved responsiveness to boost. Stolfi et al. used a stochastic agent-based immune simulation platform to explore the impact of the time interval between vaccine doses on the immunological memory elicited by a SARS-CoV-2 Spike-encoding adenoviral vaccine in humans. The computational model was calibrated to reproduce the serological results of an observational study and of a clinical trial, whereby longer intervals resulted in higher Ab responses as compared to shorter intervals (i.e., 45>20>10 weeks) (1). Stolfi et al. showed that, although the magnitude of the Ab response to boost depended on the number of pre-existing memory B cells, the difference among protocols was mainly due to Ag availability, which was reduced in the shorter interval protocols due to higher levels of pre-existing Abs and memory cytotoxic T cells. Interestingly, two groups of vaccinees emerged in the simulations, i.e., *sustainers* and *decayers*, with distinct kinetics of Ab decline due to differences in long-lived plasma cells. Repeated vaccine injections could rescue Ab levels in *decayers*, with possible implications for individuals having reduced serological memory in real life. In the simulations by Stolfi et al., memory CD8 T cells peaked after the first vaccine dose and then slowly declined, with only minor expansion after any second dose, in agreement with the few available data on human CD8 response to COVID-19 adenoviral vaccines (2). This is quite different from the experimental findings by Natalini et al. Such apparent discrepancy may derive from one or more of the following differences between the study by Stolfi et al. and that by Natalini et al.: i) a homologous versus heterologous prime/boost protocol was used; ii) model parameters were adjusted based on human vaccination results versus data obtained in mice; iii) the shortest and intermediate intervals analysed in the computational model (i.e. 10 and 20 weeks, respectively) were in the same range of the longest one in the mouse study (i.e. 100 days), and the additional interval of 45 weeks was lacking in the mouse study. Other possible differences include, for example, distinct Ag-specific naïve CD8 T cell frequencies, and diversities in Ag availability. It should be noted that the identification of similarities and discrepancies between simulations and real experiments appears greatly fruitful for further advancements in the immunological memory field (3).

## 5 Modeling memory T cell homeostasis

Swain et al. modeled *in silico* in a hypothetical laboratory mouse two classic hypotheses on the mechanisms regulating memory T cell homeostasis, i.e., ‘global’ competition for cytokines, and ‘cognate’ competition for Ag. Over time, the former led to a skewed T cell repertoire dominated by the first immune responses, while the latter to a more realistic scenario of high TCR diversity in the memory population. However, given the limited experimental evidence for ‘cognate’ competition, the authors worked on the ‘global’ competition model to improve it. They found that introducing ‘cellular aging’, along with a small continual source of memory T cells (either from stem-cell-like memory T cells or from naive T-cell recruitment into the memory pool) lead to a more convincing model of memory T cell homeostasis. The concept of ‘cellular aging’ took into account the declining cell fitness depending on the number of cell divisions, considering that there is a maximal number of divisions a somatic cell can go through, i.e., the so-called ‘Hayflick limit’. Strikingly, recent experiments in mouse models demonstrated that memory CD8 T cells specific for a single Ag were able to mount a fully functional response *in vivo* after 16 serial adoptive transfers and >50 immunizations, with no apparent limit to the number of cell divisions. The surprising results of these beautiful experiments suggested that memory CD8 T cells maintained their proliferation potential for about 10 years, a time about 3-times longer than mouse lifespan (4). Once again, experimental results and data obtained from simulations challenge each other, thus stimulating further studies, and opening new promising opportunities to improve our understanding of immunological memory.

## Author contributions

AP and FD wrote the first draft, which was integrated with insightful contributions by UH and FC. All authors approved the text for submission.
